# Supplementary figures and images for: BBK32 attenuates antibody-dependent complement-mediated killing of infectious Borreliella burgdorferi isolates
Source: PLoS Pathog. 2025 Jul 24;21(7):e1013361. doi: 10.1371/journal.ppat.1013361 (PMC12316396; doi:10.1371/journal.ppat.1013361)

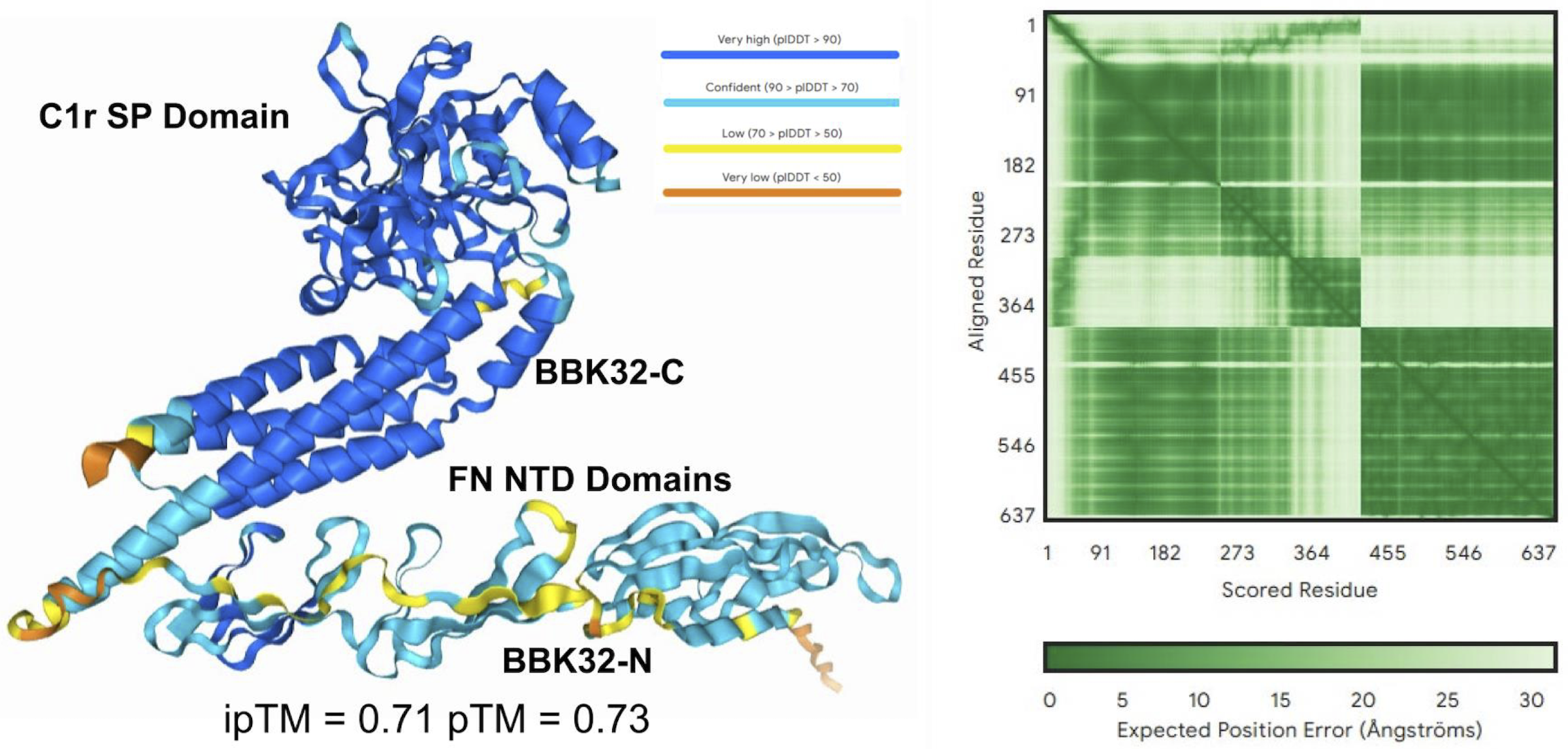

Supplement: S1 Fig — The per residue confidence metric predicted local distance difference test (pLDDT) values are shown on the model using AlphaFold3’s standard coloring scheme. pTM: Predicted template modeling score. ipTM: interface predicted template modeling score. The predicted aligned error (PAE) plot is shown on the right with BBK32 corresponding to residues 1–218, Fn to residues 219–397, and C1r residues to 398–637. (TIF) [file ppat.1013361.s001.tif]

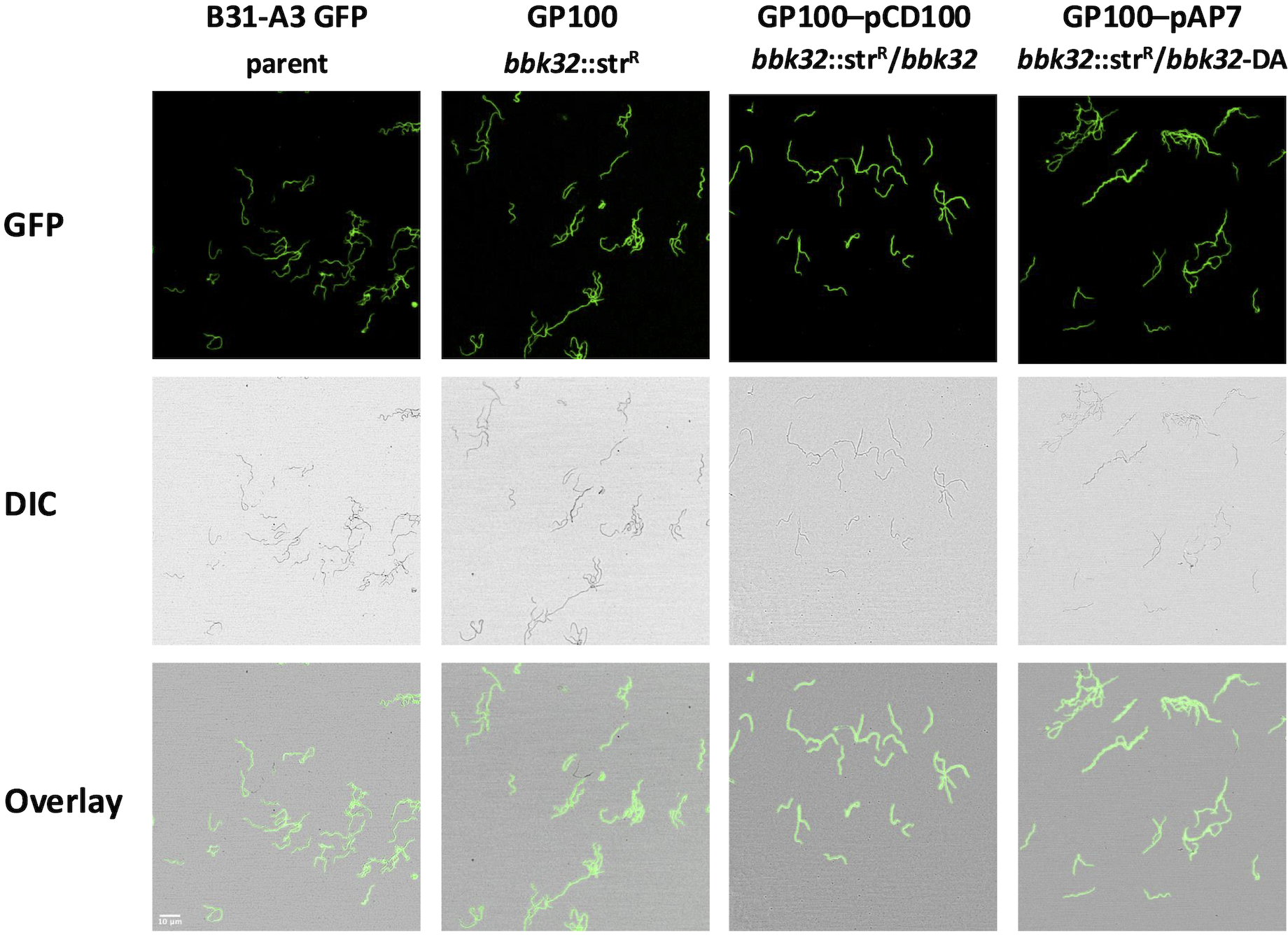

Supplement: S2 Fig — B. burgdorferi strains B31-A3 GFP, GP100 (B31-A3 GFP bbk32::StrR), GP100 pCD100 (bbk32::StrR with native bbk32 complement), and GP100 pAP7 (bbk32::StrR with bbk32-R248A/K327A [DA] complement) were fixed as previously described and imaged via confocal microscopy in the GFP and DIC channels. (TIF) [file ppat.1013361.s002.tif]

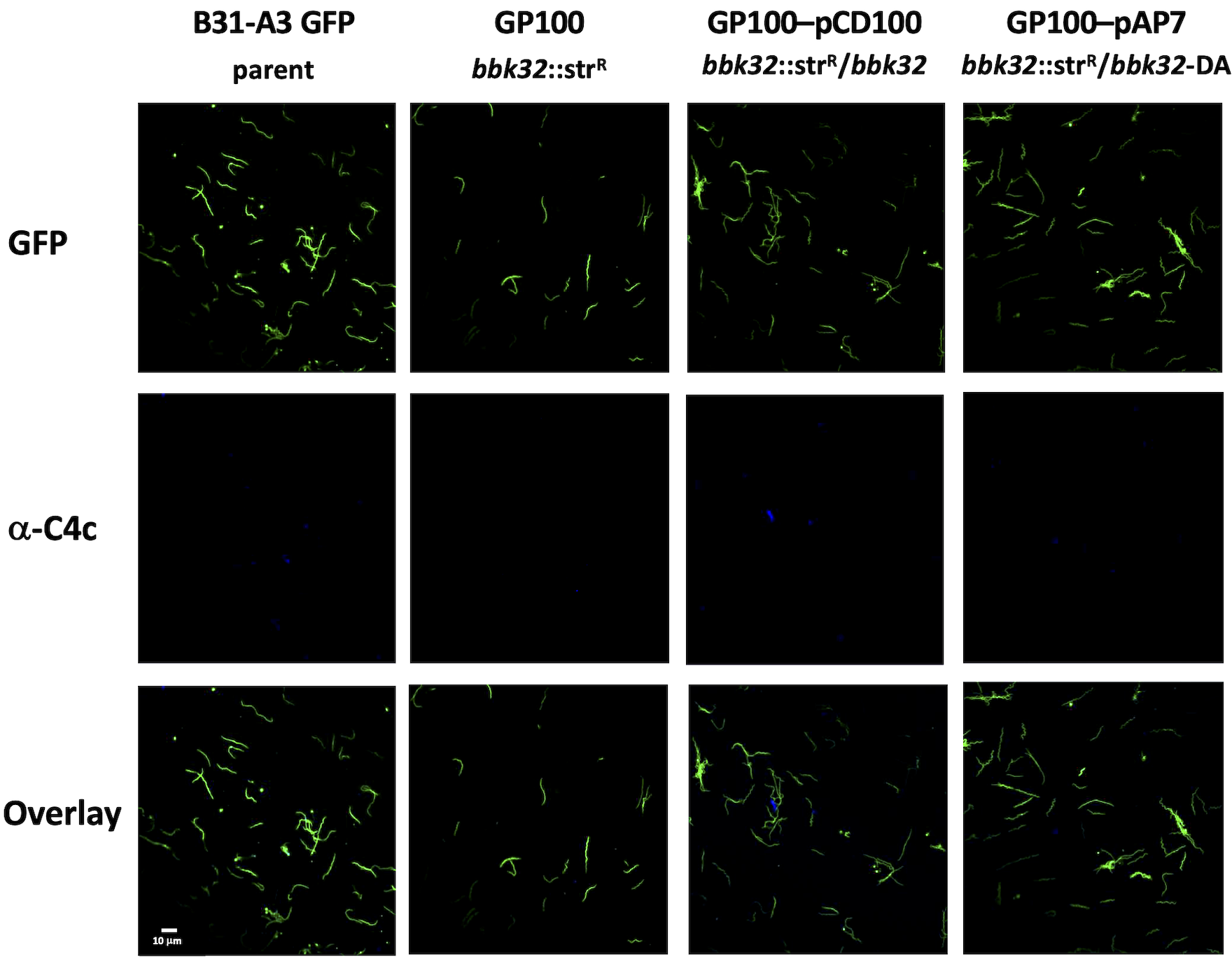

Supplement: S3 Fig — B. burgdorferi strains B31-A3 GFP, GP100 (B31-A3 GFP bbk32::StrR), GP100 pCD100 (bbk32::StrR with native bbk32 complement), and GP100 pAP7 (bbk32::StrR with bbk32-R248A/K327A [DA] complement) were incubated with an anti-rabbit isotype control antibody coupled with C5-depleted serum. Cells were then probed with murine anti-C4c, followed by anti-mouse Cy5. Cells were fluorescently imaged via confocal microscopy and the degree of rabbit isotype antibody-dependent C4c deposition was assessed. (TIF) [file ppat.1013361.s003.tif]

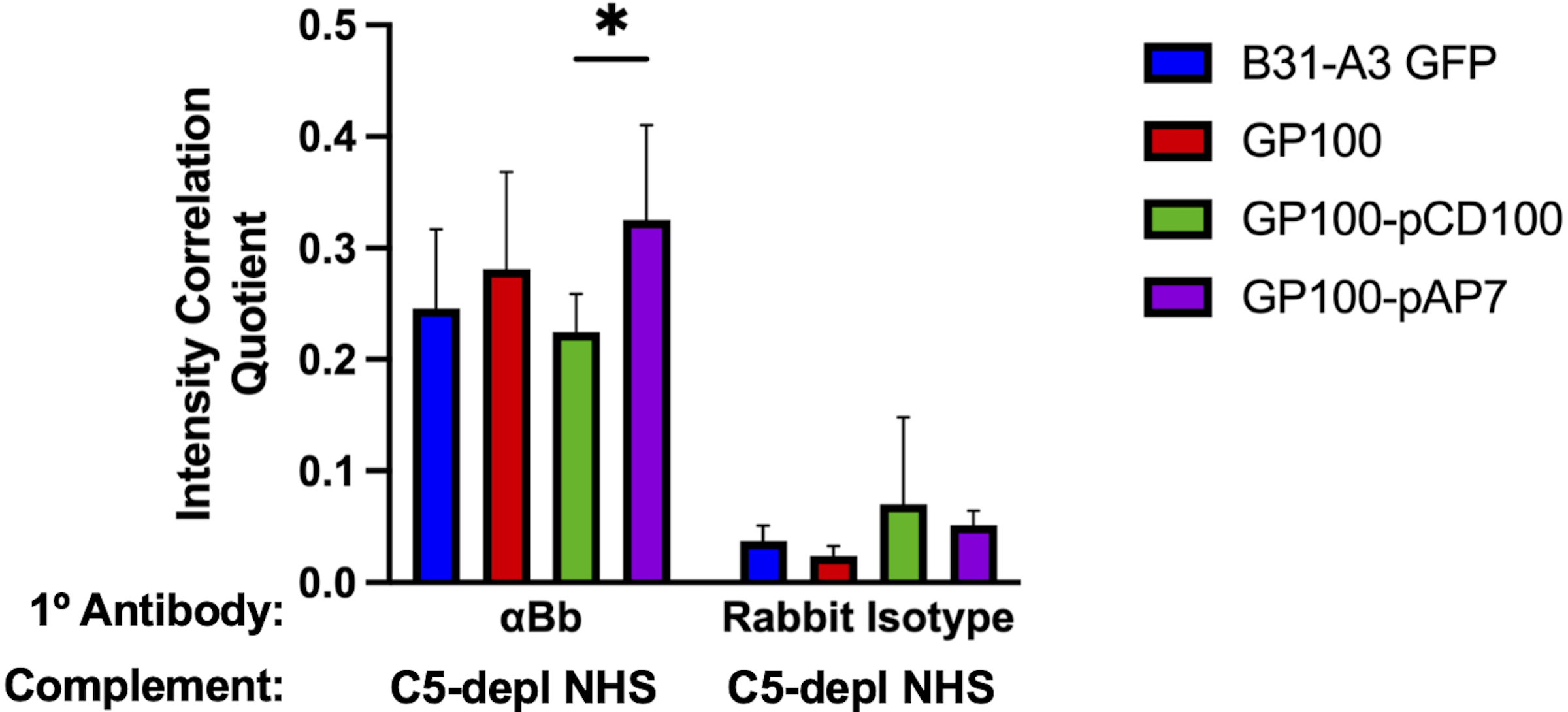

Supplement: S4 Fig — Five images of B. burgdorferi (one representative image of each group is represented in Fig 5) were scored for the colocalization of GFP and C4c using the integrated coefficient quotient (ICQ) analysis as indicated in the methods. The ICQ for each group is plotted for cells treated with C5-depleted NHS (C5-depl NHS) and either antibody against B. burgdorferi or the rabbit isotype control. * P < 0.05. (TIF) [file ppat.1013361.s004.tif]

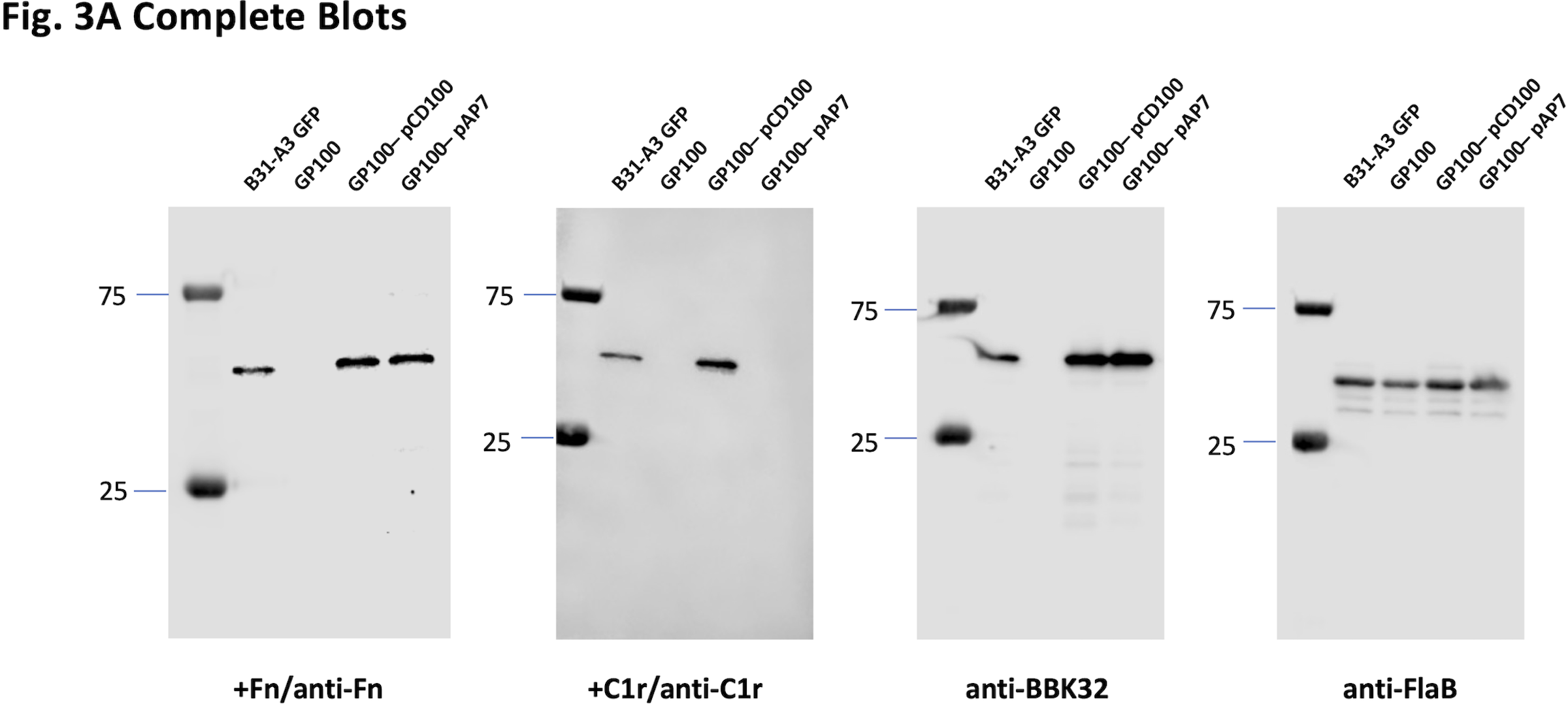

Supplement: S5 Fig — (TIF) [file ppat.1013361.s005.tif]

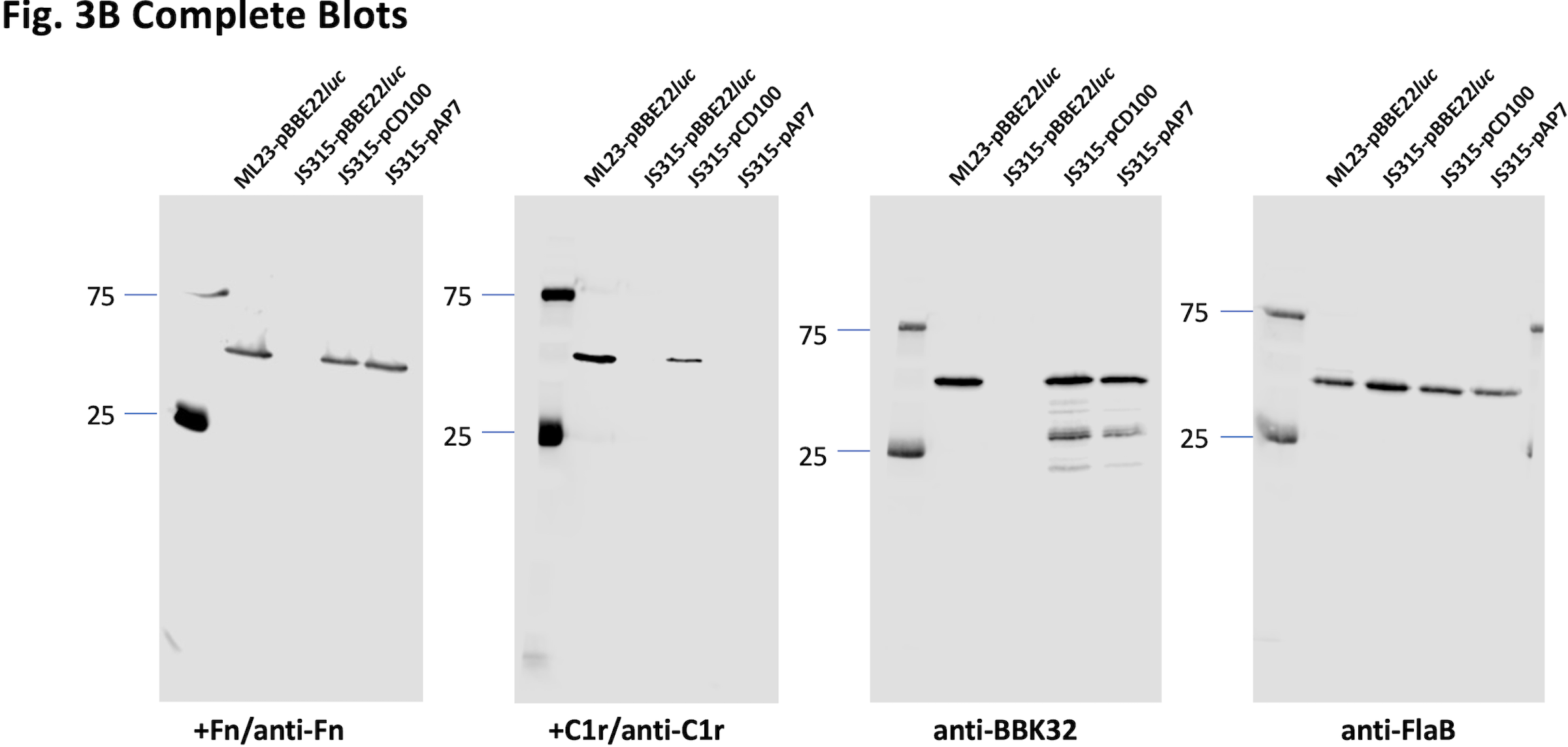

Supplement: S6 Fig — (TIF) [file ppat.1013361.s006.tif]
