# Supplementary material for: BBK32 attenuates antibody-dependent complement-mediated killing of infectious Borreliella burgdorferi isolates
Source: PLoS Pathog. 2025 Jul 24;21(7):e1013361. doi: 10.1371/journal.ppat.1013361 (PMC12316396; doi:10.1371/journal.ppat.1013361)
Supplement: Attachment — Submitted filename: PLoS_Pathogens_Powell_ADCK_Crit_Resp_250708.pdf [file ppat.1013361.s009.pdf]

Rebuttal Response to Editor and Reviewer Comments

PLOS Pathogens manuscript number: PPATHOGENS-D-25-01145
"BBK32 attenuates antibody-dependent complement-mediated killing of infectious *Borreliella burgdorferi* isolates"

We thank the Reviewers and the Academic Editor for their support of our submission. Their input—and our response to their suggestions—has resulted in an improved version of our work. We have carefully considered all the comments below and have addressed each of them individually. We are grateful for the feedback and feel confident that we have provided strong rationale for their status within the revised manuscript. Please see our responses below in blue.

Reviewers' Comments:

Reviewer's Responses to Questions

Part I - Summary
Please use this section to discuss strengths/weaknesses of study, novelty/significance, general execution and scholarship.

Reviewer #1: The team of the corresponding authors (Garcia and Skare) have published a series of papers demonstrating the anti-complement (classical pathway) functions of BBK32. In this work, their team (Powell-Pierce et al.) identified the C1r-binding sites, independent on the previously reported Fn-binding sites (activities). Leverage on this information, they further attributed this site (and BBK32-mediated C1r-binding activity) to spirochete's ability to evade antibody-mediated killing. The work was elegantly done and the paper was well written. I only have few suggestions, specifically on discussing this work and previous work to provide the physiological implications of Lyme borreliae pathogenesis.

We appreciate the support of Reviewer #1. We address the Reviewer's specific comments in Part II.

Reviewer #2: Skare et al. submitted this manuscript, BBK32 attenuates antibody-dependent complement-mediated killing of infectious Borrelia burgdorferi isolates. This study presents a wealth of new information regarding the interaction of BBK32, FN, and C1r. The authors discovered that BBK32 could bind to ECM, fibronectin, and C1r simultaneously to evade antibody-dependent complement-mediated killing. The authors further applied the AlphaFold3 analysis to predict a ternary complex of BBK32. Other techniques, such as flow cytometry, Fluorescent Microscopy, localization Analysis, and Antibody-Dependent Complement-Mediated Killing Assays, are used to delineate further the evasion mechanisms mediated by the virulence factor BBK32.

We are grateful for the supporting comments provided by Reviewer #2.

Reviewer #3: This is a well-written manuscript addressing the question as to whether BBK32, a fibronectin binding protein of Borrelia burgdorferi, can simultaneously bind fibronectin and also complement C1 without losing the capacity to interfere with complement function. This question is relevant to understanding how a single molecule can serve multiple functions to allow spirochetes to disseminate and evade host immunity in the early pathogenesis of Lyme disease. The study builds on previous structural data to predict fibronectin and complement binding sites on BBK32, and makes use of parent, mutant and reconstituted spirochete mutants to show that BBK32 can protect spirochetes from complement-mediated killing in vitro by reducing C4 deposition on spirochetes. The studies performed are sound, impact more than one Borrelia strain, and the results are clear from the data presented. While it would be helpful to show that this occurs in vivo, the multitude of pathways that Borrelia spirochetes use to evade complement-mediated killing will likely lead to inconclusive results unless these other pathways are also interrupted, which is beyond the scope of this study.

We thank Reviewer #3 for their laudatory comments regarding our submission.

Reviewer #4: This study by Powell-Pierce et al examined whether the two distinct functions previously ascribed to the Borrelia burgdorferi protein BBK32, namely binding to fibronectin and complement C1r could happen without interference from each other. Indeed, previously published identification of the relevant binding residues on the molecule (As131-162 and AS 206-354, respectively, and crystal structures of BBK32 = C1r and BBK32-Fn all had indicated that. This was then here confirmed by using alpha-fold predictions and functionally by plasmon resonance and Western blotting experiments with various mutants. In addition, the authors showed using different mutants of BBK32 that complement deposition was reduced when analyzed by flow cytometry (no primary data shown), image analysis (quantification lacking) and functionally by in vitro killing. Those last assays were done without the testing of Fn binding interference. Overall, the study confirmed their previous findings that BBK32 supports Bb survival in the presence of complement and previous crystal structure analysis and mutant analysis demonstrating that FN and Complement bind to different parts of the protein.

We thank the Reviewer for their comments. It is important to note that this study does not confirm prior survival of *Borrelia burgdorferi* in the presence of complement but extends this knowledge base by evaluating the role of BBK32 in complement resistance in infectious isolates. All prior studies were done using a serum sensitive, non-infectious, background.

Reviewer #5: This paper continues the past work, mainly by these investigators, into the functionality of the B. burgdorferi protein BBK32 as protection from complement-mediated killing following infection. In addition to the ability to bind C1r, BBK32 also has a dual property in that it binds ECM components including fibronectin. The authors investigated

two hypotheses, that BBK32 can simultaneously bind both C1r and fibronectin, and whether BBK32 provided protection against complement activation in the presence of anti-B. burgdorferi antibodies. The data presented predicts by in vitro AlphaFold3 analysis and confirmed by surface plasmon resonance that dual binding can occur. The authors proceed with experiments designed to demonstrate selective binding of C1r and fibronectin to various wild type and BBK32 mutated proteins and two B. burgdorferi parent strains by Far Westerns followed by assessments of complement activations by flow cytometry and immunofluorescent microscopy. By the results of these experiments, the authors conclude that BBK32 attenuates antibody-mediated complement killing of B. burgdorferi which had not previously been identified.

We thank the Reviewer for their positive assessment of our work here.

Part II – Major Issues: Key Experiments Required for Acceptance
Please use this section to detail the key new experiments or modifications of existing experiments that should be absolutely required to validate study conclusions.

Generally, there should be no more than 3 such required experiments or major modifications for a "Major Revision" recommendation. If more than 3 experiments are necessary to validate the study conclusions, then you are encouraged to recommend "Reject".

Reviewer #1: (No Response)

Reviewer #2:

The authors have performed SPR using the full-length FN and C1R. Since the authors have reported the BBK32 binding sites of Fn (amino acid residues 131-162) and C1r (206-354), it may be helpful to include the binding domain in the performance of SPR.
The authors may include isothermal titration calorimetry (ITC) further to prove the interaction of Bkk32 with FN and C1r, as it is a valuable technique for studying protein-protein interactions using both surface plasmon resonance (SPR) and ITC.

We thank the reviewer for this comment. Prior work by our labs, and in the labs of others, have extensively addressed the mapping of BBK32 binding sites to Fn and C1r using multiple techniques that include surface plasmon resonance, isothermal titration calorimetry, ELISA-type binding assays, and ligand blotting. Indeed, the mapping of residues 131-162 as the Fn-binding site on BBK32 was first discovered in 2001 using a gene fragment expression system and far western blotting (PMCID: PMC98480). This interaction was later confirmed by ELISA-type binding assays (PMID: 15292204 and PMCID: PMC2671840), isothermal titration calorimetry (PMID: 15737988 and PMID: 24962582),

surface plasmon resonance (PMCID: PMC3508296, PMCID: PMC4437914, and PMID: 27434228), fluorescence polarization and stopped-flow fluorescence (PMCID: PMC4412574) and x-ray crystallography (PMCID: PMC4139255). Similarly, the interaction of residues 206-354 with C1r has been validated using surface plasmon resonance, x-ray crystallography and complement functional assays across multiple studies (PMID: 26808924, PMID: 30897158, PMID: 34759015, PMID: 35693799, PMID: 37380082).

Given the rigorous nature of the prior work, that includes the suggested ITC measurements for the Fn/BBK32 interactions, we believe further data involving interaction analysis of BBK32 with each individual ligand would add little new information. However, a limitation of all of these prior studies was that the interaction with BBK32 with Fn and C1r was studied in isolation. In this work, we instead aimed to address this gap by predictive structural modeling (Fig. 1) and by devising a biophysical assay that would assess BBK32 retains the ability to interact with C1r and Fn in their biologically relevant molecular contexts (i.e., the C1 complex (*i.e.*, $C1qC1r_2C1s_2$; ~760kDa) and dimeric Fn (~500 kDa).

Reviewer #3:

The authors have provided a description of additional studies that are either in progress or would be necessary to have a more complete story of how BBk32's dual role as an adhesin and as an inhibitor of complement contributes to Borrelia burgdorferi pathogenesis. However, I believe this manuscript is a complete enough story as is and no additional experiments are necessary for this to be published.

We thank Reviewer #3 for their comments.

Reviewer #4:

1) The data in Figure 4 cannot be verified without showing the flow cytometry results.

We agree and apologize for the oversight. The data are now shown in panels A through C of Figure 4 along with the initial histogram, now shown as panel Fig. 4D.

2) The imaging results for C4c deposition on Bb requires proper image quantification to normalize for the presence of Bb. It is common to also provide a darkfield image to confirm the presence of gfp expression on all spirochetes. Indication should be provided about the number of spirochetes analyzed and the frequency of those showing deposition. If deposition is reduced but not eliminated, then an average MFI might be used instead.

We have considered this point very carefully and have provided scoring data that addresses the signal from a collection of each strain tested (5 images per strain). The package used is the JACOP intensity correlation coefficient-based analysis plugin for ImageJ. This analysis looks for the degree of colocalization of signals from the images and scores them for their intensity correlation quotient (ICQ) analogous to MFI. When this was

done, the data showed that the *bbk32* mutant strain and the *bbk32* mutant transformed with the *bbk32*-R248A/K327A double mutant (that cannot bind or inhibit C1r) deposit more C4c on the surface of GFP-producing *B. burgdorferi*. This contrasts what is observed for the parent and the bbk32 mutant complemented with the wildtype allele of *bbk32* where the C4c signal is reduced. The differences observed were only significant between the wildtype and *bbk32*-R248A/K327A double mutant complements but the cells with wildtype BBK32 did exhibit lower levels of C4c deposition consistent with the hypothesis that BBK32 reduces antibody-mediated complement activation. This is admittedly a qualitative assessment that we followed up in Figure 6 with quantitative data showing significant differences in damage to cells with functional versus impaired BBK32. We also conducted this analysis with cells that used a non-borrelial isotype antibody control. *B. burgdorferi* cells incubated with the isotype control showed little to no colocalization consistent with a lack of complement activation due to little or no antibody recognition (supplemental Figure 3). The ICQ data for the anti-*B. burgdorferi* and isotype control incubation are shown in supplemental Figure 4. Regarding the request to evaluate the production of GFP by all spirochetes, we have submitted a new supplemental figure (Supplemental Fig. 2) showing that all spirochetes exhibit fluorescence by merging the *B. burgdorferi* GFP image with the same field using DIC microscopy. Although not shown in the initial submission, in our hands this strain stably produces GFP in all *B. burgdorferi* observed.

3) It is unclear why figures 4-6 were done exclusively testing of complement inhibition, which had been previously amply demonstrated, when the novel information to be provided in this manuscript was to explore the interaction of Fn-C binding simultaneously.

The analysis reported here is distinct from any of our prior reports. In this manuscript we use antibody specific to *B. burgdorferi* in infectious isolates with or without functional BBK32. Our prior work used a serum sensitive strain of *B. burgdorferi* (strain B314; lacks all linear plasmids) with different forms of BBK32 or BBK32-like proteins to assess resistance to classical complement using normal human serum. As such, the data shown in Figures 5 and 6 have never been reported in any way previously and demonstrate that BBK32 does partially protect infectious *B. burgdorferi* from some degree of antibody-dependent, complement-mediated killing.

Reviewer #5: No major issues

Part III – Minor Issues: Editorial and Data Presentation Modifications
Please use this section for editorial suggestions as well as relatively minor modifications of existing data that would enhance clarity.

Reviewer #1:

1. Before the authors' team identified BBK32 as a C1r-binding protein (Garcia et al. PLoS Pathog 2016), BBK32 was known to have two functions (glycosaminoglycan (GAG)-binding activity and fibronectin (Fn)-binding activity). Lin et al. Cell Microbiol 2015 from Dr. John Leong's team and Moriarty et al. Mol. Microbiol. 2012 from Dr. George Chaconas's lab located where those activities are on the BBK32 (residues 45-68 and 158 to 182 (and 182 to 209) of BBK32). Lin et al. and Moriarty et al. have used the spirochetes producing BBK32 with internally deletions on the amino acids 45-68 and 158-182 to attribute Fn- and GAG-functions of BBK32 to vascular interactions (through short term i.v. injection of mice) and early stages joint colonization (through low dose, intradermal inoculation of mice). Therefore, the information described in the line 115-116 and 433-434 may not be accurate, especially to reflect the findings of those papers.

We thank the reviewer for these points that they raise. We have modified both statements to acknowledge the comments made and to also clarify what we meant, particularly in the latter issue raised. Briefly, while it is true that in frame deletions have been tested for BBK32 that reduce or abrogate GAG or Fn binding, we favor using forms of full length BBK32 that are mutated in important contact residues. This approach should reduce large structural changes that might have indirect negative effects and thus confound interpretation.

2. The authors have identified BBK32 to be able to simultaneously bind to Fn and C1r, suggesting that the functions of Fn-binding and C1r-binding of BBK32 can be performing simultaneously during infection. GAG binds to amino acids 45-68 of BBK32, far from Fn and C1r-binding sites. Therefore, although the authors did not characterize whether BBK32 can bind to Fn, C1r, simultaneously with GAG, it is very likely that BBK32 can bind to these three ligands simultaneously. As Lin et al. 2015 and Moriarty et al. 2012 have defined the functions of GAG and Fn for BBK32 during infection, the physiological implications in the case that BBK32 evolved to bind to three ligands at the same time would be worth to discuss. Lin et al. and Moriarity et al. each proposed a model to explain the functions of GAG and Fn at early stages of infection. C1r seems to be the last piece of puzzle of the picture to elucidate the role of BBK32 during early infection.

This is valid point raised by the Reviewer. We have added an additional sentence in the Discussion to indicate that this possibility is likely but will require additional experimentation in future studies (also see the response to the prior comment).

3. The papers from one of the corresponding authors in this manuscript, Dr. Skare, (Seshu et al. Mol. Microbiol. 2006 and Hyde et al. Mol. Microbiol. 2011) showed that a bbk32 mutant (the strain JS315 in ML23 missing lp25) when introduced into the mice under low dose close to ID50 (10^3 per mouse) displayed partial colonization defects at early stages of infection. This work later was later confirmed by this manuscript and Lin et al. Cell Microbiol. 2015, indicating the strength of controlling the dose under intradermal injection to delineate the roles of functionally redundant Lyme borreliae proteins. However, Li et al. from Dr. Erol Fikrig's laboratory showed that a bbk32 mutant in the background of a

bacterial strain with lp25 does not have any defects in mouse-to-tick acquisition, tick-to-mouse transmission, and tissue colonization (Li et al. Infect Immun 2006). It would be worth to discuss these results of BBK32 from the perspectives of Lyme borreliae pathogenesis. Such discussions would be helpful because it has been very common for the strain deficient of different Lyme borreliae gene (e.g., DbpA, CspZ, CspA) showing different phenotypes under tick vs. needle infection models.

While we respect the opinion of the Reviewer, we have opted not to address this as we believe citing the Li et al. work will be more confounding than illuminating. The only comparison between the Li work and our prior work (Seshu et al., 2006) is the large inoculum dose at 10^5 *B. burgdorferi* that they tested for their parent, mutant, and complement strains. Note that the 10^5 dose was the **only** needle inoculum they tested. We also tested that amount for the *bbk32* mutant (along with parent and complement) and, like them, saw no phenotype. Only at a smaller inoculum dose of 10^3 *B. burgdorferi* did we see a phenotype for the loss of *bbk32*. Other than pointing out this differential, which we have done in other papers (see Hyde et al., 2011), there seems to be limited value to addressing this issue. Regarding their tick infectivity analysis, there are many examples of borrelial mutant strains with phenotypes in infected mice that can colonize ticks effectively. As such, for the sake of space, we have not elaborated on this work here.

4. The authors mentioned that some partial phenotypes in this study could be due to the compensatory functions of Elp (line 476-477). Does the background strain that is in this study encode Elp?

Yes, the wildtype strains used have been confirmed to carry cp32-1, cp32-7, cp32-9, lp28-1, and lp56, which encode *elpB, elpM, elpQ, elpD*, and *elpX*, respectively. As such, these strains will encode additional proteins that have been shown to inhibit the classical pathway of complement. In unpublished data, we have shown that the CRISPRi based knock down of *elpQ* reduces resistance to antibody-dependent complement mediated killing in *B. burgdorferi* upon selective induction of a guide RNA specific for *elpQ*. If the same construct is induced in a *bbk32* mutant, the sensitivity to antibody-dependent complement mediated killing increases significantly. We have indicated this in the current manuscript as we feel it is currently being developed independently and beyond the scope of this study.

Reviewer #2:

Fig. 3: It would be helpful to have a band density plot using ImageJ. The present legend is not easy to understand. It may be useful to provide a brief explanation in the legend.

After considering this comment, we do not think that the ImageJ analysis would provide useful information for what we are showing in Figure 3. These are chemiluminescent blots, and the signal observed may not be on a linear scale when bands between strains are measured by ImageJ on a given blot. If this is the case (likely), the actual amount between

samples might not be quantified accurately. Secondly, the intent of this figure is to address the ability of *B. burgdorferi*—via functional BBK32 protein--to either bind fibronectin (Fn) or C1r. The answer we are scoring is either a "yes" or a "no". All the samples, other than the *bbk32* mutant, bind Fn. However, only strains with the native BBK32 sequence are capable of recognizing C1r. Specifically, while the BBK32-R248A/K327A double alanine (DA) mutant can bind to Fn, it does not bind to human C1r, consistent with earlier biochemical and functional assays we have previously published for the DA mutant form of BBK32.

Fig. 5: It may be helpful to include an arrow in the overlay and provide a brief explanation in the legend.

We thank the Reviewer for this suggestion but think that an arrow would have limited value since there is detectable colocalization in all panels. Instead, we are including a means to integrate colocalized signal in Supplemental Figure 4 that shows the GFP signal with enhanced C4c levels. We feel that this—along with the improved images in Figure 5—alleviate the issue here, making the use of an arrow unnecessary.

Reviewer #3:
This manuscript is clearly written. The only modification suggested is in the Discussion section, lines 394-397, regarding the stated hypothesis that survival of B. burgdorferi after exposure to Borrelia antibodies and complement would be diminished in the presence of functional BBK32. If this is indeed the hypothesis, then there should be another sentence indicating that their results are opposite to this due to the ability of BBK32 to bind both fibronectin and inhibit complement activation simultaneously.

We thank the Reviewer for catching this error on our end. We have fixed this issue to indicate that the presence of BBK32 "enhances" borrelial survival (as intended).

Reviewer #4:

1) Studies by John Weis (Jacobson et al. 2007) and others showed that complement receptor-mediated killing is not required for control of Borrelia by mice. Thus, the extent to which complement can opsonize to support Borrelia-killing is unclear. A point that should be considered and acknowledged.

While we appreciate the extensive work of our colleagues' using mice that are deficient in various complement components in conjunction with *B. burgdorferi* infection, including CR1 and CR2, the intent of our work is focused on the classical complement pathway where the organisms are most likely subject to clearance by the formation of the terminal membrane attack complex (MAC) independent of these host receptors. One of the most prominent phenotypes for any of the mouse complement knockouts linked to *B. burgdorferi* infection is the work we published using C1q$\alpha^{-/-}$ mice (Zhi et al., 2018). In this work we showed that the infectious load of *B. burgdorferi* is increased when C1q is absent,

consistent with a role for classical complement in limiting borrelial infection. Surprisingly, other mouse complement component knockouts (in factor H, C3, C5, and CR1/CR2) have limited to no clear phenotype following infection with *B. burgdorferi*. Because of the complexity inherent to these comparisons, and the uncertainty associated with their linkage with our current in vitro data using human serum, we believe detailed discussion of prior work involving complement knockout mice (including CR1/CR2) would likely detract from the results presented herein. As such, we have left this discussion out of the revised manuscript and have cite a book chapter and review co-authored by our groups at appropriate points throughout that better address these studies in the context of Lyme disease pathogenesis (PMC8046170 and PMC7572514).

Reviewer #5:

1. As a personal opinion, I prefer the Supplemental Figures 3 and 4 over Figure 3. Showing the entire blots rather than selective cutaways has always seemed more believable (at least to me). Plus, the blots are beautifully clean from non-specific binding. Just a note for consideration.
Another small note; the authors could have considered adding a recombinant BBK32 lane to the blots to ensure that the fibronectin and C1r were indeed binding to native BBK32 in the lysate lanes instead of the possibility of binding to a different protein that co-migrates with BBK32 in the gel.

We thank the Reviewer for their suggestion. However, we have decided to keep the arrangement from the initial submission with the smaller frames for Figure 3 and the full blots for the supplemental Figures (now Supplemental Figures 5 and 6 in the revised manuscript.

For the second comment, we believe that the addition of recombinant BBK32 would have minimal benefit given that we have addressed any background issue with the isogenic *bbk32* mutant tested for all the Westerns and overlays done. All blots were run on the same gel system and utilized the same separation conditions. We also used a monoclonal antibody to BBK32 that is extremely sensitive and specific. The overlays line up perfectly with the BBK32 Western. Furthermore, and more importantly, the isogenic mutant that lacks BBK32 shows no signal for Fn or C1r binding under the conditions of our experimental set up. Altogether, this provides strong rationale to not add recombinant BBK32 as an additional control to our blots.

2. Regarding the fluorescent microscopy images in Fig. 5, hopefully it would be possible to lighten the brightness on the images especially for the anti-C4c pictures. Even on the computer screen, it was difficult to compare those images with the overlay images. So the statement in the text Results lines 357-362 that more C4c was seen in the mutant strains than the parents doesn't look convincing and is debatable. Perhaps lightening the images and adding arrows to show borrelia without C4 staining in the parents could be helpful. The flow data from Fig. 4 is more convincing.

We have adjusted the images—equivalently for all samples—to address the Reviewer's concern. Also, see the comments under Part II, Reviewer #3, comment #2 for further input into the issue raised here by the Reviewer.

3. Strictly an editorial comment. The authors provide a comprehensive background narrative in the Introduction and the Discussion. However, although nicely structured and informative, the entire text (mainly in the Results) could be improved for reading by eliminating needless words and phrases. There are several instances of: "to (or towards) this end…", "in order …", "…we then…" , "to begin…", "we sought to…"; " we next…" that when removed streamlines the reading.

We appreciate the Reviewer's advice here. We have gone through the manuscript and streamlined the text, removing many of the phrases referred to here.

Line 69; "these hosts" may be better than "organisms"

Done as specified.

Line 102; "pathways" (plural)

Thank you to the Reviewer for this catch. We have changed this as indicated.

Lines 107-109; sentence repeats "classical pathway". Could be rewritten as " BBK32, which is upregulated during vertebrate infection, can potently block activation of the classical pathway of complement by binding to C1r ".

We agree with the Reviewer's assessment and have incorporated this change.

Lines 277-279; this sentence redundant to line 272 that starts this paragraph. Can be omitted.

We omitted the redundant sentence as suggested.
